# Serum uric acid: A risk factor for right ventricular dysfunction and prognosis in heart failure with preserved ejection fraction

**DOI:** 10.3389/fendo.2023.1143458

**Published:** 2023-03-06

**Authors:** Xiang-liang Deng, Han-wen Yi, Jin Xiao, Xiao-fang Zhang, Jin Zhao, Min Sun, Xue-song Wen, Zhi-qiang Liu, Lei Gao, Zi-yang Li, Ping Ge, Qi Yu, Dong-ying Zhang

**Affiliations:** ^1^ Department of Cardiovascular Medicine, The First Affiliated Hospital of Chongqing Medical University, Chongqing, China; ^2^ Department of Cardiovascular Medicine, The First Branch of the First Affiliated Hospital of Chongqing Medical University, Chongqing, China

**Keywords:** serum uric acid, right ventricular dysfunction, heart failure with preserved ejection fraction, hyperuricemia, prognosis

## Abstract

**Background:**

Hyperuricemia and right ventricular dysfunction (RVD) are both widespread in heart failure with preserved ejection fraction (HFpEF) patients. RVD is associated with a poor prognosis in HFpEF. The correlation between serum uric acid (UA) levels and right ventricular function is unclear. The prognostic performance of UA in patients with HFpEF needs further validation.

**Methods and results:**

A total of 210 patients with HFpEF were included in the study and divided into two groups according to UA level: the normal UA group (≤7 mg/dl) and the high UA group (>7 mg/dl). The variables examined included clinical characteristics, echocardiography, and serum biochemical parameters. Right ventricular function was assessed by tricuspid annular plane systolic excursion (TAPSE) and tricuspid annular peak systolic velocity (TAPSV). Baseline characteristics were compared between the two groups, and the correlation between baseline UA and RVD was assessed using multifactorial binary logistic regression. Kaplan–Meier curves were used to describe all-cause mortality and heart failure readmission. Results showed that right ventricular function parameters were worse in the high UA group. After adjusting for UA, left ventricular posterior wall thickness (LVPWT), N-terminal B-type natriuretic peptide (NT-proBNP), atrial fibrillation (AF), and low-density lipoprotein cholesterol (LDL-C), UA (odds ratio = 2.028; *p* < 0.001) was independently associated with RVD, and UA >7 mg/dl (HR = 2.98; *p* < 0.001) was associated with heart failure readmission in patients with HFpEF.

**Conclusion:**

Elevated serum UA is closely associated with RVD and significantly associated with the heart failure readmission rate in patients with HFpEF.

## Introduction

Heart failure with preserved ejection fraction (HFpEF) is generally considered a syndrome with pathophysiological heterogeneity, whose prevalence has increased rapidly over the past two decades (1, 2). Factors affecting the prognosis (mortality and hospitalization) of HFpEF include metabolic syndrome, renal insufficiency, and so forth (3). Hyperuricemia is an important comorbidity in heart failure patients and is usually associated with advanced severity of heart failure (4).

As the end product of purine metabolism in the human body, uric acid (UA) is commonly associated with the development and progression of cardiovascular diseases such as peripheral artery disease, coronary artery disease (CAD), hypertension, and atrial fibrillation (AF) (5–7). The prevalence of hyperuricemia ranges from 13.4% to 20.1% in different populations (8–10), and the total number of people with hyperuricemia in China has gradually increased to 170 million (11). Elevated UA was particularly common in people with heart failure in China (12), and previous studies have shown that hyperuricemia may contribute to worse clinical outcomes in patients with cardiovascular diseases (13, 14).

Right ventricular dysfunction (RVD) is one of the common manifestations in the HFpEF population (15). Former studies have demonstrated that RVD leads to a worse clinical prognosis compared to HFpEF patients without RVD. However, the current treatment of RVD has not been as effective as anticipated (16–18). This study aimed to investigate the relationship between UA and RVD in the context of HFpEF and to illustrate the relationship between UA and the prognosis of HFpEF.

## Patients and methods

### Study design and study population

**Figure 1:**
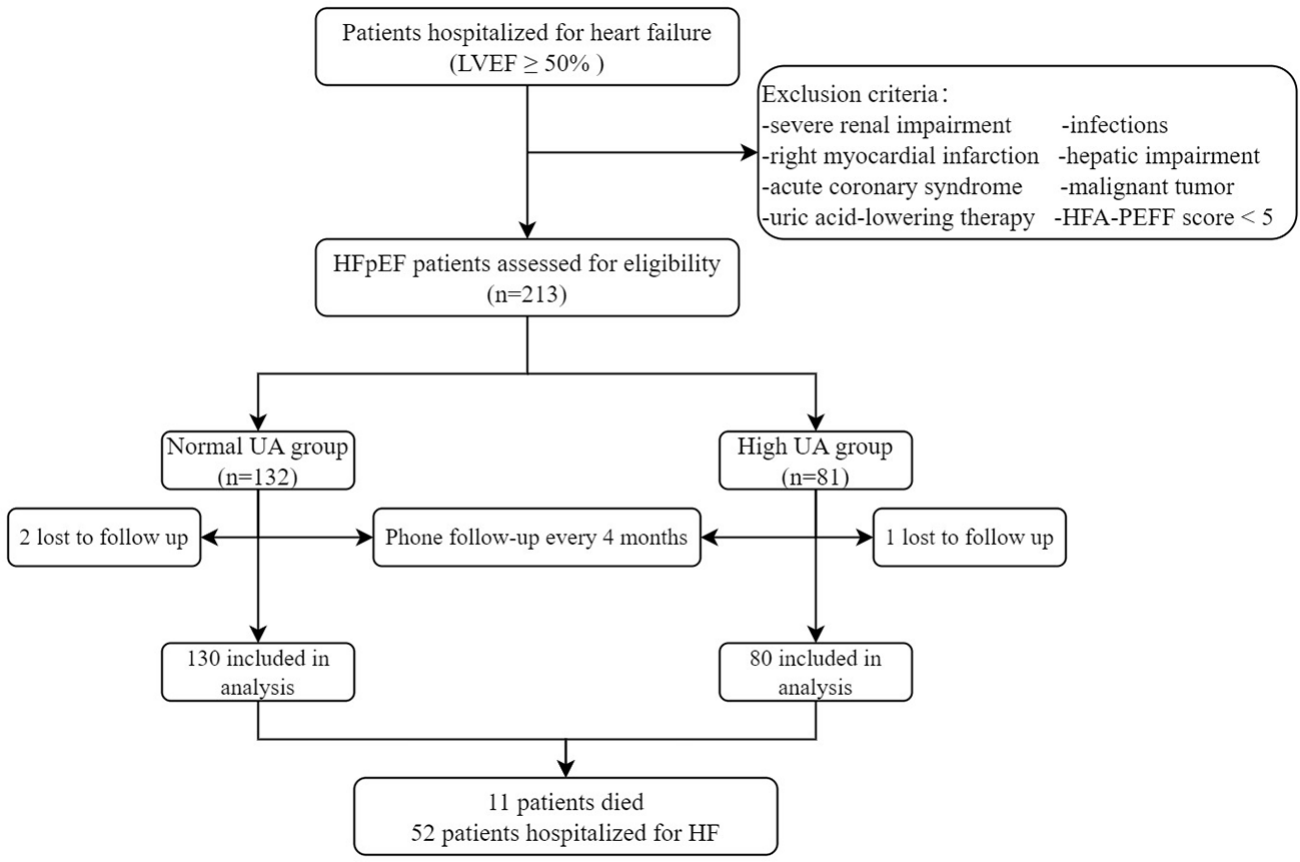
The enrollment flowchart. HFpEF, heart failure with preserved ejection fraction; HF, heart failure; LVEF, left ventricular ejection fraction; TAPSE, tricuspid annular plane systolic excursion; TAPSV, tricuspid annular peak systolic velocity; UA, uric acid.

This is a prospective observational study to assess the association between baseline UA and RVD in patients with HFpEF and to investigate the relationship between elevated UA and patient prognosis. Study patients were enrolled between October 2020 and April 2022. All enrolled patients met the inclusion criteria for a definitive diagnosis of HFpEF according to the HFA-PEFF diagnostic algorithm. The exclusion criteria were (1) acute coronary syndrome or right myocardial infarction history, (2) severe renal impairment (eGFR < 30 ml/min/1.73 m^2^, based on CKD-EPI formula), (3) urate-lowering therapy, (4) malignant tumor, (5) severe hepatic impairment (elevated liver enzymes: three times over upper reference limit or liver cirrhosis), and (6) infections. According to Chinese guidelines for the diagnosis and management of hyperuricemia and gout in 2019, hyperuricemia is defined as above 7.0 mg/dl (19). Patients enrolled in the study were divided into two groups: normal UA group (UA ≤7.0 mg/dl) and high UA group (UA >7.0 mg/dl). All the study population signed informed consents that were prospectively registered and agreed to be followed up for the collection of outcome data. Patients were followed up by phone every 4 months, and three patients were lost during the follow-up period. The last follow-up visit ended in August 2022. Ultimately, 210 patients were enrolled in the study (**Figure 1**). The study was in accordance with the Declaration of Helsinki, approved by the Clinical Research Review Board of the First Affiliated Hospital of Chongqing Medical University (No. 2021-473), and registered on clinicaltrials.gov with an identifier of NCT05053256.

### Data collection

The baseline clinical data collection was conducted by trained researchers following the same protocol at the time of enrollment. Patients’ demographics, comorbidities, personal histories, medications, laboratory tests, and echocardiography were collected. Biochemical indexes were detected and analyzed, including albumin (Alb), blood urea nitrogen (BUR), creatinine (Cr), direct bilirubin (DB), hemoglobin (Hb), glycosylated hemoglobin (HbAlc), high-sensitivity C-reactive protein (hs-CRP), LDL-C, N-terminal B-type natriuretic peptide (NT-proBNP), and UA.

### Serum uric acid measurement

Blood samples were taken on the second morning after admission. UA was performed through the central laboratory using ABBOTT. Conversion of each UA measurement from micromoles per liter to milligrams per deciliter was conducted by dividing it by 60.

### Echocardiography and assessment of right ventricular function

All the echocardiographic examinations were conducted by trained echocardiographers according to the guidelines of the American Society of Echocardiography (ASE) (20). The standard four-chamber method was used to measure the right atrial transverse diameter, right ventricular anteroposterior diameter, tricuspid annular plane systolic excursion (TAPSE), and tricuspid annular peak systolic velocity (TAPSV). We defined RVD as TAPSE <17 mm and TAPSV <9.5 cm/s. Pulmonary systolic pressure (PASP) was calculated as 4 * (peak tricuspid regurgitation velocity (TR))^2^ + right atrial pressure, estimated based on the diameter and collapse of the inferior vena cava.

### Outcomes and clinical follow-up

Endpoints examined include readmission for heart failure and all-cause mortality. Heart failure readmission was determined by two senior doctors in the heart failure ward. Deaths were confirmed by population management consultations and hospital death certificates. Enrolled patients were followed up by telephone or WeChat every 4 months until the end of August 2022 or death. Postcharge clinical events were obtained through telephone follow-up and medical records from other hospitals.

### Statistical analysis

We used percentages for qualitative data. Normally distributed quantitative data were presented as mean ± standard deviation (SD), and abnormally distributed quantitative data were presented as median (interquartile range (IQR)). The receiver operating characteristic (ROC) curve was used to determine the predictive value of UA for RVD. When comparing baseline data for HFpEF patients with UA > 7 mg/dl and UA ≤ 7 mg/dl, independent sample *t*-test, rank sum test, or Chi-square test were selected based on data characteristics. Pearson’s or Spearman’s tests were used to assess the association of variables with UA, TAPSE, and TAPSV. Based on published data and clinical relevance, we performed a univariate analysis of UA, gender, CAD, diabetes, AF, body mass index (BMI), systolic blood pressure (SBP), heart rate, left ventricular posterior wall thickness (LVPWT), and interventricular septal thickness (IVST). Based on the results of univariate binary logistic analysis, different models were developed to determine the odds ratio (OR) between UA and RVD. The long-term cumulative incidence of all-cause mortality and heart failure readmission was estimated using Kaplan–Meier curves. The predictive value of variables for heart failure readmission was tested by Cox’s univariate proportional hazards regression analysis. Variables in univariate Cox regression were included in multivariate Cox regression, and subgroup analysis was performed to demonstrate the potential effects of UA >7 mg/dl. Statistical significance was *p* < 0.05. Results were expressed as a hazard ratio (HR) with 95% confidence intervals (95% CI). All statistical analyses were performed using SPSS version 24.0 (SPSS, Chicago, IL, USA).

## Results

### Clinical characteristics

In total, 210 eligible HFpEF patients were recruited to the study (59% women), with 80 patients assigned to the high UA group. Compared to the normal UA group, patients in the high UA group were more prevalent with CAD, AF, and higher New York Heart Association (NYHA) class heart failure, higher SBP, diastolic blood pressure (DBP), heart rate, BUR, Cr, NT-proBNP, DB, and hs-CRP, but lower Alb and LDL-C at baseline (*p* < 0.05; **Table 1**). Except for the higher frequency of diuretics use in the high UA group, there was no difference in medication use or other characteristics, including age, BMI, HbAlc, history of smoking, or alcohol consumption between the two groups (**Table 1**).

**Table 1:** Baseline characteristics of the study population according to serum uric acid.

	Total (*n* = 210)	Normal UA (≤7 mg/dl, *n* = 130)	High UA (>7 mg/dl, *n* = 80)	*p*-value
Demographics				
Age (years)	74 (67, 81)	72 (67, 79)	77 (66, 82)	0.132
Gender/men (*n*, %)	86 (41.0%)	45 (34.6%)	41 (51.2%)	0.017
BMI (kg/m^2^)	23.6 (21.1, 26.4)	23.6 (21.5, 25.9)	23.6 (20.6, 26.7)	0.668
SBP (mmHg)	125 ± 15.6	121 ± 12.5	132 ± 17.5	<0.001
DBP (mmHg)	73 ± 9.7	70 ± 7.7	77 ± 11.1	<0.001
Heart rate	76 (66, 89)	74 (65, 85)	84 (71, 98)	0.001
Comorbidities				
CAD (*n*, %)	94 (44.8%)	45 (34.6%)	49 (61.3%)	<0.001
Hypertension (*n*, %)	136 (64.8%)	83 (63.8%)	53 (66.3%)	0.723
Diabetes (*n*, %)	74 (35.2%)	49 (37.7%)	25 (31.3%)	0.343
AF (*n*, %)	84 (40.0%)	39 (30.0%)	45 (56.3%)	<0.001
NYHA class				
II	106 (50.5%)	81 (62.3%)	25 (31.3%)	<0.001
III	92 (43.8%)	46 (35.4%)	46 (57.5%)	0.002
IV	12 (5.7%)	3 (2.3%)	9 (11.3%)	0.007
Personal history				
Smoking (*n*, %)	54 (25.7%)	31 (23.8%)	23 (28.7%)	0.430
Drinking (*n*, %)	41 (19.5%)	20 (15.4%)	21 (26.3%)	0.054
Medications				
Diuretics (*n*, %)	141 (67.1%)	78 (60%)	63 (78.8%)	0.005
ACEI/ARB/ARNI (*n*, %)	132 (62.9%)	83 (63.8%)	49 (61.3%)	0.705
Statins (*n*, %)	158 (75.2%)	103(79.2%)	55(68.8%)	0.087
Laboratory values				
UA (mg/dl)	6.14 (5.05, 7.60)	5.24 (4.67, 5.92)	8.10 (7.48, 9.12)	<0.001
BUR (mmol/L)	6.8 (5.6, 9.1)	6.4 (5.3, 7.9)	8 (6.1, 9.9)	<0.001
Cr (µmol/L)	79 (63, 96)	69 (60, 85)	94 (79, 115)	<0.001
NT-proBNP (pmol/L)	114.8 (52.8, 262.4)	83.3 (50.5, 164.7)	220.6 (109.3, 344.1)	<0.001
hs-CRP (mg/L)	2.03(0.74, 6.94)	1.40 (0.67, 4.69)	2.91 (1.39, 9.79)	0.001
Hb (g/L)	130 (119, 143)	128 (120, 141)	131 (120, 146)	0.177
LDL-C (mmol/L)	2.13 (1.63, 2.59)	2.20 (1.71, 2.64)	1.97 (1.46, 2.47)	0.020
TB (µmol/L)	11.9 (8.4, 17.6)	11.0 (8.0, 14.4)	14.4 (9.5, 20.7)	0.001
DB (µmol/L)	5.1 (3.4, 7.2)	4.4 (3.2, 6.0)	6.2 (4.4, 9.9)	<0.001
Alb (g/L)	40 (38, 43)	42 (39, 44)	39 (37, 41)	<0.001
HbAlc (%)	5.6 (6, 6.4)	6.0 (5.6, 6.4)	5.9 (5.7, 6.4)	0.687
Echocardiography				
TAPSE (mm)	17.9 ± 3.7	18.9 ± 3.3	16.2 ± 3.7	<0.001
TAPSV (cm/s)	10.5 (9.0, 12.8)	11.6 (10.2, 13.2)	9.1 (7.9, 9.8)	<0.001
PASP (mmHg)	42 (34, 49)	39 (32, 45)	45 (36, 56)	0.002
RA diameter (mm)	39 (35, 45)	37 (33, 42)	43 (37, 50)	<0.001
RV diameter (mm)	21 (20, 24)	20 (19.22)	22 (20, 26)	<0.001
LA diameter (mm)	36 (32, 41)	36 (32, 40)	38 (32, 42)	0.133
LVEDD (mm)	47 ± 5.9	46 ± 5.8	47 ± 6.2	0.264
LAVI (ml/m^2^)	40 (33, 54)	40 (33, 53)	44 (33, 56)	0.147
LVMI (kg/m)	112 (96, 140)	113 (96, 140)	111(96, 140)	0.894
LVPWT (mm)	10.0 (10.0, 11.0)	10.0 (10.0, 11.0)	10.0 (10.0, 12.0)	0.723
IVST (mm)	11.0 (10.0, 12.0)	11.0 (10.0, 12.0)	10.5 (10.0, 12.0)	0.979
LVEF (%)	61 (58, 65)	62 (58, 65)	60 (57, 64)	0.108
H2FPEF score	4 (3, 5)	3 (3, 5)	5 (3, 6)	0.004
HFA-PEFF score	6 (5, 6)	6 (5, 6)	6 (5, 6)	0.088
RVD (*n*, %)	72 (34.3%)	19 (14.6%)	53 (66.3%)	<0.001

Values are mean ± standard deviation, number (%), or median (interquartile range). AF, atrial fibrillation; Alb, albumin; BMI, body mass index; BUR, blood urea nitrogen; CAD, coronary artery disease; Cr, creatinine; DB, direct bilirubin; DBP, diastolic blood pressure; HbA1c, glycosylated hemoglobin; Hb, hemoglobin; hs-CRP, high-sensitivity C-reactive protein; IVST, interventricular septal thickness; LA, left atrium; LAVI, left atrial volume index; LDL-C, low-density lipoprotein cholesterol; LVEDD, left ventricular end-diastolic dimension; LVEF, left ventricular ejection fraction; LVPWT, left ventricular posterior wall thickness; LVMI, left ventricular mass index; NT-proBNP, N-terminal B-type natriuretic peptide; PASP, pulmonary artery systolic blood pressure; RV, right ventricular; RVD, right ventricular dysfunction; SBP, systolic blood pressure; TAPSE, tricuspid annular plane systolic excursion; TAPSV, tricuspid annular peak systolic velocity; TB, total bilirubin; UA, uric acid.

The echocardiographic characteristics of patients are presented in **Table 1**. Compared with the normal UA group, patients in the high UA group displayed worse right heart structure and function, including larger right ventricular (RV) diameter and right atrium (RA) diameter, higher PASP, and lower TAPSE and TAPSV, but similar left ventricular end-diastolic dimension (LVEDD), left atrial volume index (LAVI), left ventricular mass index (LVMI), and left ventricular ejection fraction (LVEF). In the present study, the prevalence of RVD was 34.3% among all participants, and the prevalence in the high UA group was four times higher than that in the normal UA group (66.3% vs. 14.6%; *p* < 0.001; **Table 1**).

### The association among UA, TAPSE, TAPSV, and selected variables

Spearman’s correlations among UA, TAPSE, TAPSV, and selected variables are summarized in **Table 2**. UA was positively correlated with PASP, heart rate, SBP, Cr, NT-proBNP, hs-CRP, and DB (*p* < 0.05) but negatively correlated with TAPSE, TAPSV, LDL-C, and Alb (*p* < 0.05). In addition, TAPSE and TAPSV had positive associations with IVST, LVPWT, LDL-C, and Alb, but were negatively associated with PASP, heart rate, Cr, NT-proBNP, hs-CRP, and DB (*p* < 0.05; **Table 2**). The correlation between UA and echocardiographic characteristics representing right heart dysfunction is indicated in **Supplementary Figure S1**.

**Table 2:** Correlation analysis among UA, TAPSE, TAPSV, and other variables.

	TAPSE		TAPSV		UA	
	*r*	*p*-value	*r*	*p*-value	*r*	*p*-value
TAPSE (mm)	–	–	0.711	<0.001	−0.441	<0.001
TAPSV (cm/s)	0.711	<0.001	–	–	−0.495	<0.001
UA (mg/dl)	−0.441	<0.001	−0.495	<0.001	–	–
PASP (mmHg)	−0.290	<0.001	−0.249	<0.001	0.250	<0.001
LVPWT (mm)	0.216	0.002	0.187	0.007	−0.010	0.867
IVST (mm)	0.209	0.001	0.160	0.016	−0.050	0.581
Age (year)	−0.113	0.104	−0.164	0.018	0.088	0.203
Heart rate	−0.230	0.001	−0.193	0.005	0.214	0.002
SBP (mmHg)	0.081	0.242	0.005	0.940	0.277	0.001
LDL-C (mmol/L)	0.202	0.003	0.225	0.001	−0.155	0.025
Creatinine (µmol/L)	−0.165	0.017	−0.245	<0.001	0.514	<0.001
hs-CRP (mg/L)	−0.205	0.005	−0.151	0.040	0.255	<0.001
NT-proBNP (pmol/L)	−0.473	<0.001	−0.473	<0.001	0.381	<0.001
DB (µmol/L)	−0.343	<0.001	−0.327	<0.001	0.339	<0.001
Alb (g/L)	0.217	0.002	0.195	0.005	−0.297	<0.001

Alb, albumin; hs-CRP, high-sensitivity C-reactive protein; IVST, interventricular septal thickness; LDL-C, low-density lipoprotein cholesterol; LVPWT, left ventricular posterior wall thickness; NT-proBNP, N-terminal B-type natriuretic peptide; PASP, pulmonary artery systolic blood pressure; SBP, systolic blood pressure; TAPSE, tricuspid annular plane systolic excursion; TAPSV, tricuspid annular peak systolic velocity; UA, uric acid.

### ROC curve for the prediction of RVD

**Figure 2:**
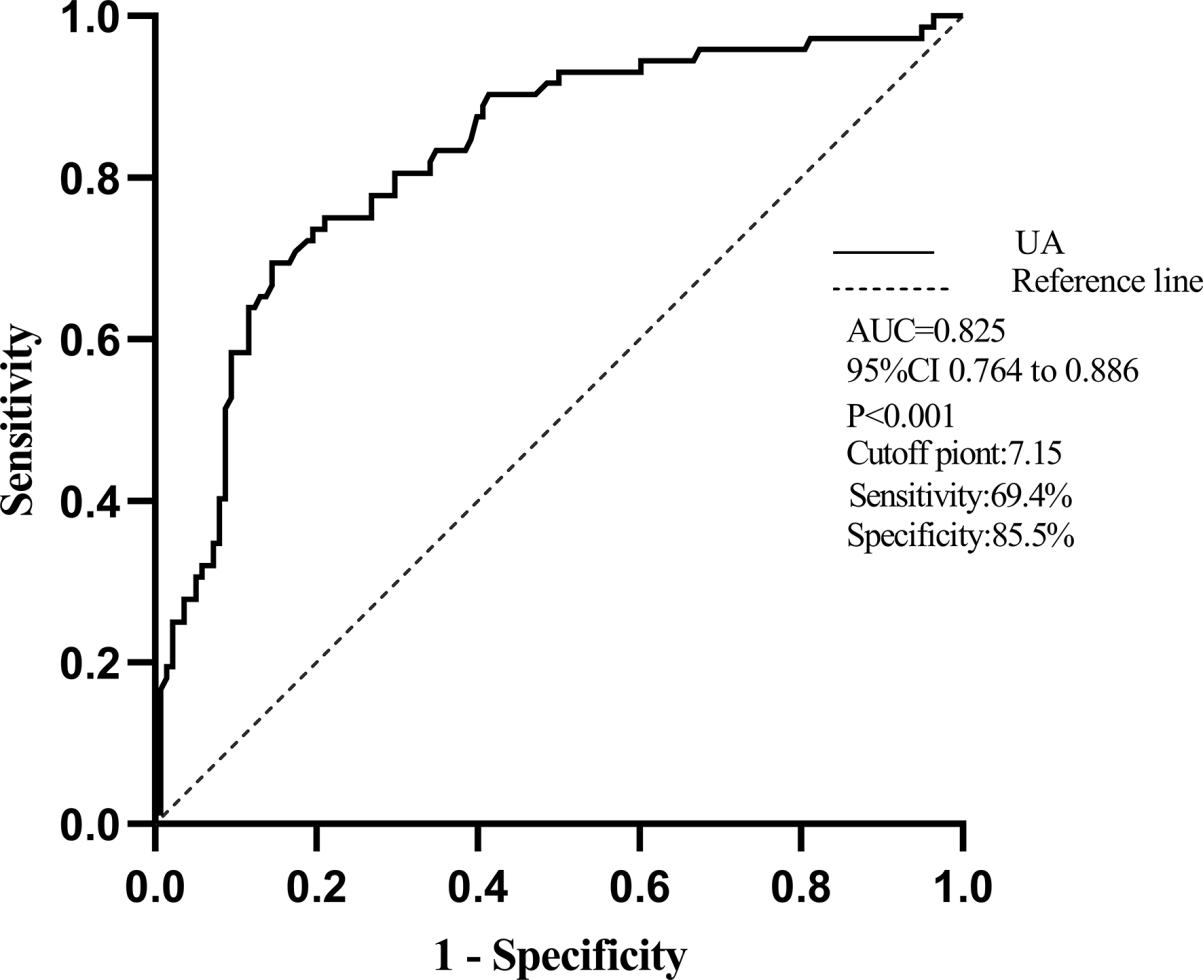
The receiver operator characteristic (ROC) curve of UA for predicting RVD. Notes: The area under the curve (AUC) for RVD was 0.825(95% CI, 0.764–0.886). The best cutoff of serum UA to predict RVD was 7.15 mg/dl with a sensitivity of 69.4% and a specificity of 85.5%.

ROC curves are shown in **Figure 2** to demonstrate the diagnostic UA value for the prediction of RVD, which was defined as TAPSE < 17 mm and TAPSV < 9.5 cm/s. The area under the curve (AUC) for RVD was 0.825 (95% CI, 0.764–0.886; *p* < 0.001). The best cutoff value of UA for predicting RVD was 7.15 mg/dl, yielding sensitivity and specificity of 69.4% and 85.5%, respectively (**Figure 2**).

### Univariate and multiple logistic regression analysis with RVD

In univariate binary logistic regression analysis, RVD was significantly associated with UA (OR = 2.061; 95% CI, 1.654–2.568; *p* < 0.001), AF (OR = 3.508; 95% CI, 1.933–6.366; *p* < 0.001), IVST (OR = 0.807; 95% CI, 0.658–0.990; *p* = 0.040), LVPWT (OR = 0.742; 95% CI, 0.593–0.928; *p* = 0.009), and other biochemical indexes including NT-proBNP, Cr, DB, and Alb (**Table 3**).

**Table 3:** Univariate binary logistic regression on the absolute value of right ventricular dysfunction.

	OR	95% CI	*p*-value
UA (mg/dl)	2.061	1.654–2.568	<0.001
Gender (*n*)	1.244	0.699–2.216	0.458
CAD (*n*)	1.068	0.603–1.893	0.822
AF (*n*)	3.508	1.933–6.366	<0.001
Diuretics (*n*)	3.099	1.555–6.179	0.001
SBP (mmHg)	0.992	0.974–1.011	0.397
Heart rate	1.023	1,009–1.037	0.001
LVPWT (mm)	0.742	0.593–0.928	0.009
IVST (mm)	0.807	0.658–0.990	0.040
Cr (µmol/L)	1.021	1.010–1.032	<0.001
NT-proBNP (pmol/L)	1.006	1.004–1.008	<0.001
hs-CRP (mg/L)	0.999	0.992–1.006	0.711
LDL-C (mmol/L)	0.538	0.362–0.800	0.002
DB (µmol/L)	1.174	1.084–1.271	<0.001
Alb (g/L)	0.842	0.774–0.916	<0.001

Alb, albumin; CAD, coronary artery disease; Cr, creatinine; DB, direct bilirubin; hs-CRP, high-sensitivity C-reactive protein; IVST, interventricular septal thickness; LDL-C, low-density lipoprotein cholesterol; LVPWT, left ventricular posterior wall thickness; NT-proBNP, N-terminal B-type natriuretic peptide; UA, uric acid.

**Figure 3:**
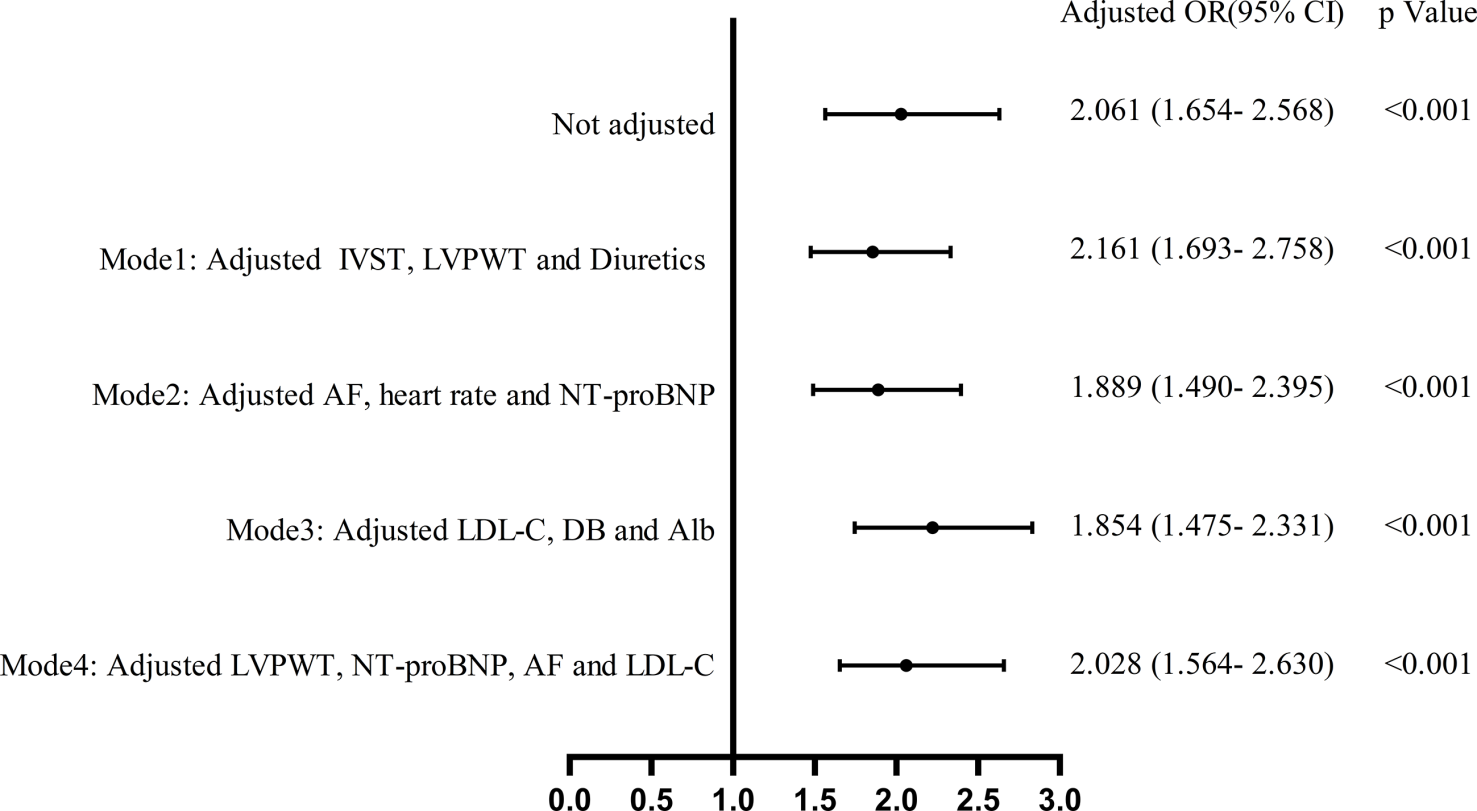
Multivariate binary logistic regression analysis of the effect of UA on the absolute value of right ventricular dysfunction.

Multivariable binary logistic regression analysis was performed using variables that were significant in univariate binary logistic regression, and four separate models were developed by the stepwise regression analysis method. Variables of the same type or related were included in one model. Variables that were significant in the first three models were taken into model 4. The results showed that UA was independently associated with RVD in all models (**Figure 3**). Details of the OR and *p*-value are listed in **Supplementary Table S1**.

### The correlation between UA and the prognosis of HFpEF

**Figure 4:**
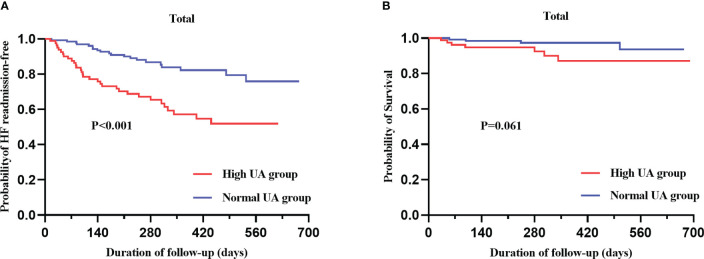
Kaplan–Meier analysis for heart failure readmission **(A)** and all-cause mortality **(B)** categorized by serum UA level.

During a median follow-up period of 278 (190–443) days, 52 (24.8%) patients were readmitted for heart failure, and 11 (5.2%) patients died. Kaplan–Meier curves for heart failure readmission and all-cause mortality are displayed in **Figure 4**. The rate of heart failure readmission was higher in the high UA group (*p* = 0.001) compared to the normal UA group, and all-cause mortality also trended to be higher without statistical significance (*p* = 0.062). The rate of heart failure readmission was higher in both male (*p* = 0.002) and female (*p* = 0.018) patients in the high UA group (**Supplementary Figure S2A**). To better understand the effect of UA on heart failure readmission, univariate and multivariate Cox regression analyses were performed. In univariate Cox regression, high UA (>7 mg/dl), RVD, and NT-proBNP were related to heart failure readmission (*p* < 0.05). Indicators in univariate Cox regression were taken into multivariate Cox regression, and high UA (HR = 3.027; *p* = 0.002) and NT-proBNP (HR = 1.002 for 1 pmol/L increase; *p* = 0.01) were independently related to heart failure readmission rate after adjusting of high UA, gender, NYHA class, CAD, AF, RVD, NT-proBNP, and Cr (**Supplementary Table S2**). To gain further insight into the role of UA in patients with HFpEF, a subgroup analysis was also conducted. The results indicated that UA > 7 mg/dl might be a risk factor for heart failure readmission regardless of gender, NT-proBNP, TAPSE, PASP, LVPWT, Alb, and DB values (**Supplementary Figure S3**). The association between high UA and heart failure readmission was stronger in male than in female patients (male (HR 3.88) and female (HR 2.43) patients).

## Discussion

The results of this prospective cohort analysis showed that UA levels were associated with the adverse change in right ventricular function in HFpEF patients, with RVD measured by TAPSE and TAPSV. Patients in the high UA group had a higher rate of heart failure readmission, but no statistical difference in all-cause mortality between the two groups was detected.

The European Society of Cardiology (ESC) guidelines highlight UA measurement as an additional marker for the stratification of cardiovascular risk (21), although hyperuricemia is common in patients with chronic heart failure with a prevalence of 50% (22), in HFpEF (26%) (23), and in our research (38%). Limited data are available regarding the relationships of UA levels in HFpEF, especially between UA and right ventricular function. In the present study, we found that hyperuricemia was independently associated with right ventricular function in HFpEF. There are few reports about whether UA correlates with right ventricular function in patients with HFpEF, but some information from previous studies suggests that correlations may exist. A previous study in asymptomatic patients with type 2 diabetes demonstrated an independent relationship between UA and biventricular systolic function, regardless of renal function or diabetic control (24). In patients with idiopathic pulmonary artery hypertension, higher UA levels were suggested to be associated with a lower cardiac index and higher pulmonary vascular resistance (25, 26). Another study in patients with ischemic heart disease or dilated cardiomyopathy showed hyperuricemia was associated with elevated right atrial pressures (27). According to those reports and our results, UA levels might correlate with pulmonary artery, right atrial pressure, and right ventricular function.

Variables frequently used to assess RV function in patients with heart failure include RV ejection fraction, the longitudinal strain of the RV, TAPSE, and TAPSV. In the current study, TAPSE and TAPSV were used to assess right ventricular function, which is recommended by ASE to improve the accuracy of RVD (20). They are negatively associated with PASP and TR, the latter two being used in HFpEF diagnosis by the diagnostic algorithm of the HFA-PEFF (28, 29). The relationship between TAPSE and UA has rarely been reported. In our study, we declared high UA levels were significantly related to lower TAPSE, which is consistent with previous reports in type 2 diabetes patients (24), suggesting that UA might be a biomarker of RVD in patients with HFpEF.

The mechanisms to account for elevated UA and RVD in HFpEF patients appear to be unclear and remain to be elucidated. The possible explanations for the findings are as follows: Firstly, increased UA production due to the upregulation of xanthine oxidase and decreased UA excretion owing to lactic acid accumulation and reduced renal perfusion resulted in a high prevalence of hyperuricemia in heart failure patients (30, 31). Secondly, animal and cell experiments have demonstrated that elevated UA may lead to an increase in cytokine activation, insulin resistance, and oxidative stress, impairing endothelial function and activating the renin–angiotensin system (32–35), which may promote pulmonary vascular remodeling. Meanwhile, the right ventricle is very sensitive to increased pulmonary vascular resistance (36). Increased PASP promotes right ventricular remodeling. Further studies are still needed to demonstrate whether UA evolves in the development of RVD or is only a risk factor for RVD in HFpEF patients.

Previous studies in China did not report gender differences in UA levels in HFpEF patients, so we grouped patients according to Chinese hyperuricemia guidelines: the normal UA group (UA ≤ 7.0 mg/dl) and the high UA group (UA > 7.0 mg/dl). However, the enrolled population showed that gender differences in UA levels did exist. In addition, several studies have shown that the effect of gender on the prognosis of HFpEF is still controversial (37–39). In our study, we performed a gender-specific adjusted analysis and found that hyperuricemia was associated with heart failure readmissions in all patients, more prominently in men. This is in line with the higher comorbidity burden in male heart failure patients (40, 41). For our results, it is important to carefully consider the gender difference in UA levels and the impact of hyperuricemia on the clinical outcomes of patients with HFpEF.

It is widely accepted that UA is an independent predictor of heart failure morbidity and worse outcomes in heart failure population (42–44). However, previous studies demonstrated that the relationship between UA and all-cause mortality or cardiovascular death was controversial both in HFpEF and heart failure with reduced ejection fraction (HFrEF). In the DAPA-HF trial, UA per 1 mg/dl unit increase was not associated with cardiovascular death (HR, 1.06 (0.99–1.14); *p* = 0.07) or all-cause mortality (HR, 1.03 (0.97–1.1); *p* = 0.25) in patients with HFrEF (45). In the EMPEROR-reduced trial, serum UA was an independent predictor of increased mortality (all-cause and cardiovascular mortality) and hospitalization for heart failure when the highest serum UA tertile was compared to the lowest serum UA tertile (4). In addition, the PARAGON-HF trial and the RELAX trial displayed inconsistent results regarding the relationship between elevated UA and all-cause mortality in HFpEF (23, 46). Multiple studies in HFrEF have shown that UA-lowering treatment with benzbromarone or allopurinol failed to improve clinical outcomes, exercise capacity, quality of life, and left ventricular systolic function (47, 48). Research in patients with hyperuricemia and HFpEF showed that UA was a predictor for the composite of all-cause mortality and HF rehospitalization and that lowering UA may improve prognosis (49). This suggests that UA-lowering therapy might help improve the prognosis of the patient. To date, there has been no study on the relationship between UA-lowering treatment and RV function in HFpEF. Therefore, whether reducing UA would improve RV function in patients with HFpEF need to be demonstrated by designing specific studies.

## Limitations

Our study has the following drawbacks: First, the sample size is limited, and the follow-up time is not long enough. Second, we did not collect the dynamic changes of UA and evaluate the prognostic effects of UA reduction. Third, improvement or deterioration of RVD was not assessed during follow-up, so the effect of UA on changes in RVD outcomes could not be obtained. Fourth, as a prospective observational study, we cannot evaluate the potential role of UA in the development and progression of HFpEF.

## Conclusion

Overall, elevated UA levels are associated with RVD in HFpEF patients and may be related to heart failure readmission in patients with HFpEF.

## Data availability statement

The raw data supporting the conclusions of this article will be made available by the authors, without undue reservation.

## Ethics statement

The studies involving human participants were reviewed and approved by Clinical Research Review Board of the First Affiliated Hospital of Chongqing Medical University. The patients/participants provided their written informed consent to participate in this study. Written informed consent was obtained from the individual(s) for the publication of any potentially identifiable images or data included in this article.

## Author contributions

X-LD contributed to the study design, data analysis, and manuscript preparation. H-WY, JX, X-FZ, JZ, MS, X-SW, Z-QL, LG, Z-YL, and PG were involved in the acquisition of data. D-YZ and QY worked on the study concept, design, and final proof. All authors read and approved the final manuscript.
