# Supplementary material for: Serum uric acid: A risk factor for right ventricular dysfunction and prognosis in heart failure with preserved ejection fraction
Source: Front Endocrinol (Lausanne). 2023 Mar 6;14:1143458. doi: 10.3389/fendo.2023.1143458 (PMC10025558; doi:10.3389/fendo.2023.1143458)
Supplement: Supplementary file 1 [file DataSheet_1.docx]

**Figure S1.**


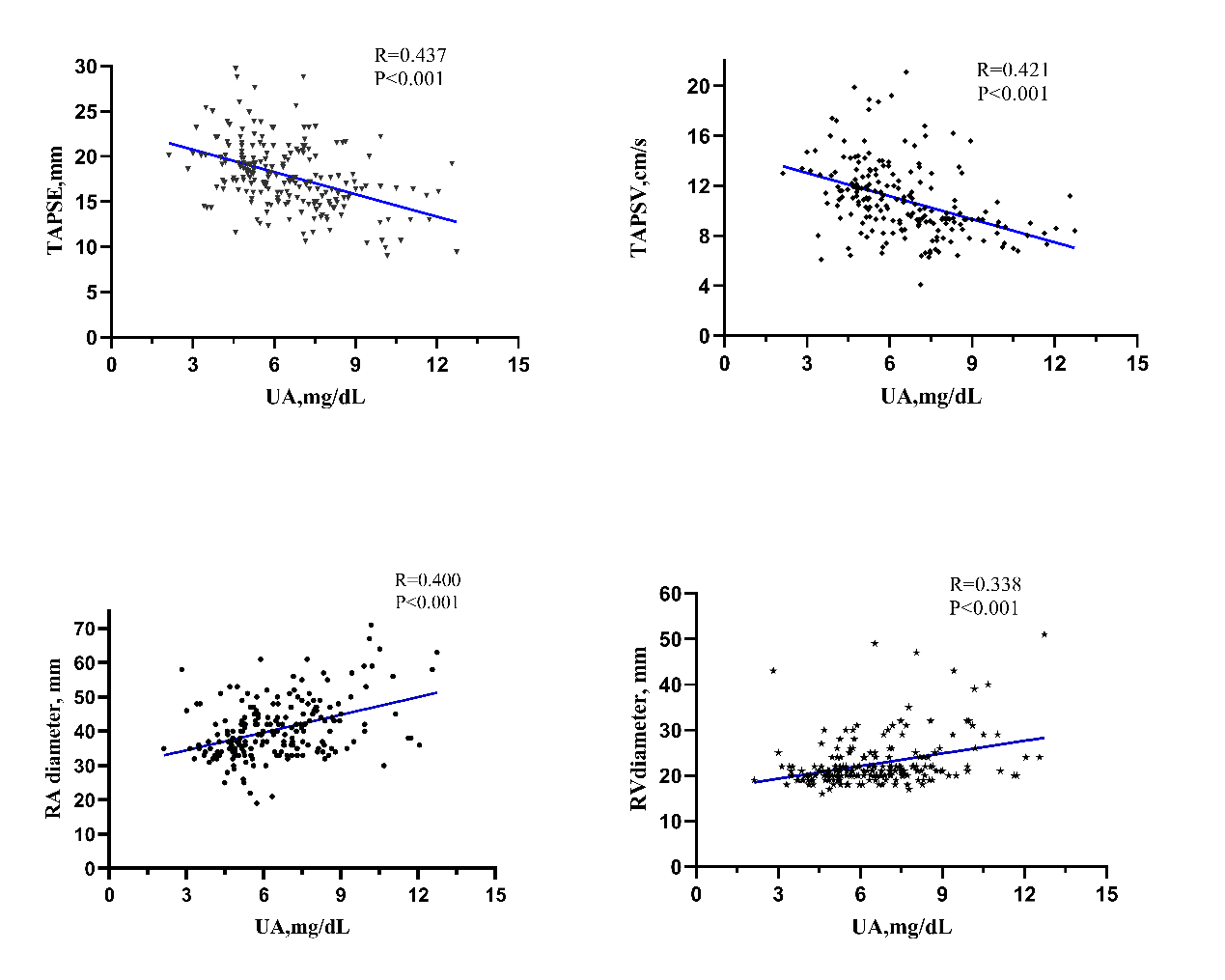


**Figure S1**. Scatter plots evaluating the association between baseline UA levels and various echocardiographic indices.

**Figure S2**

**A**


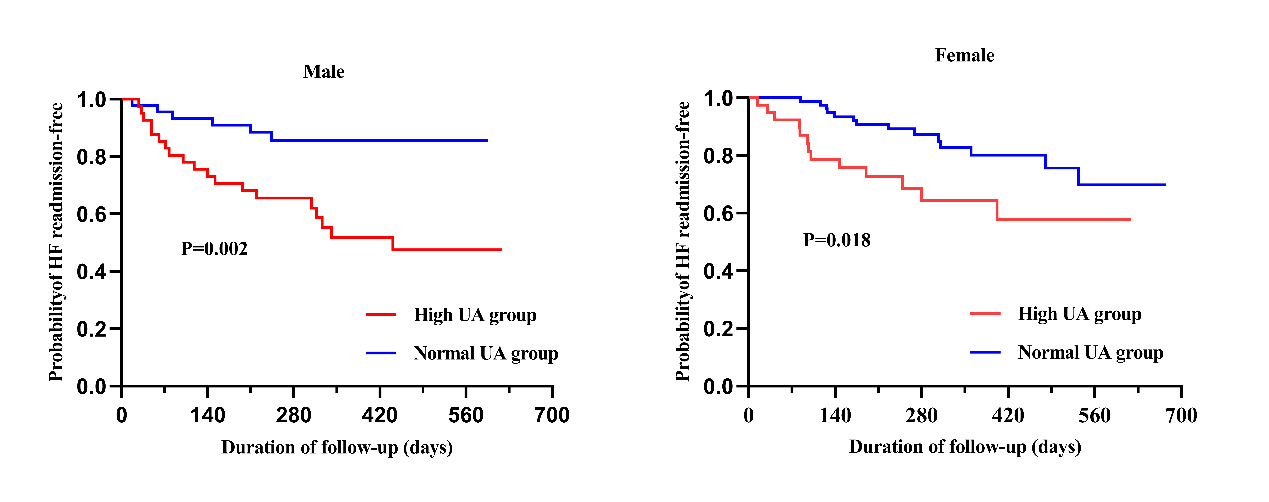


**B**

**
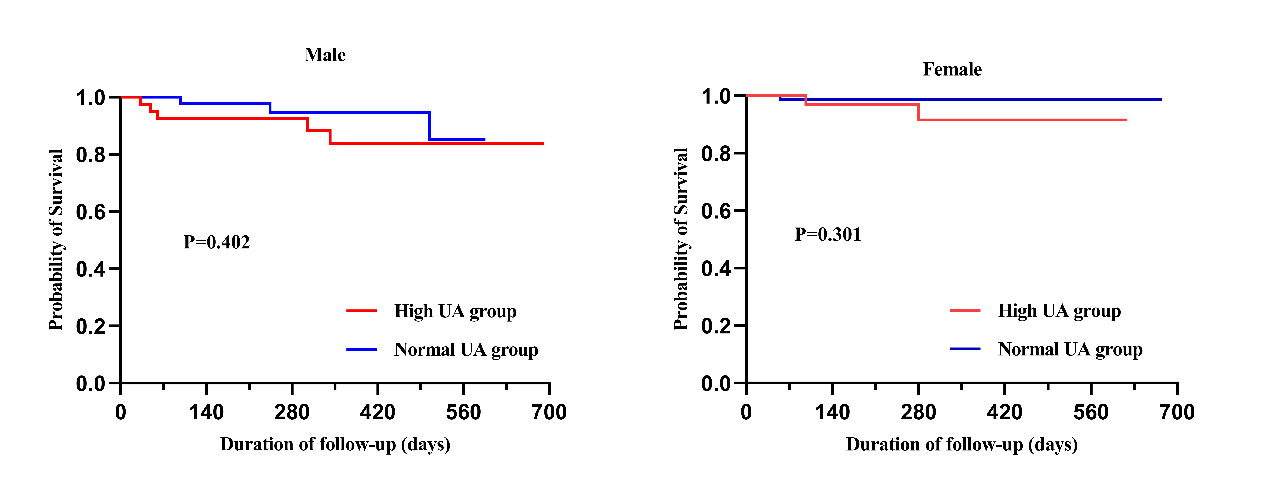
**

**Figure S2.** Kaplan-Meier analysis of heart failure readmission (A) and all-cause death (B) categorized by serum UA level according to gender.

**Figure S3**.


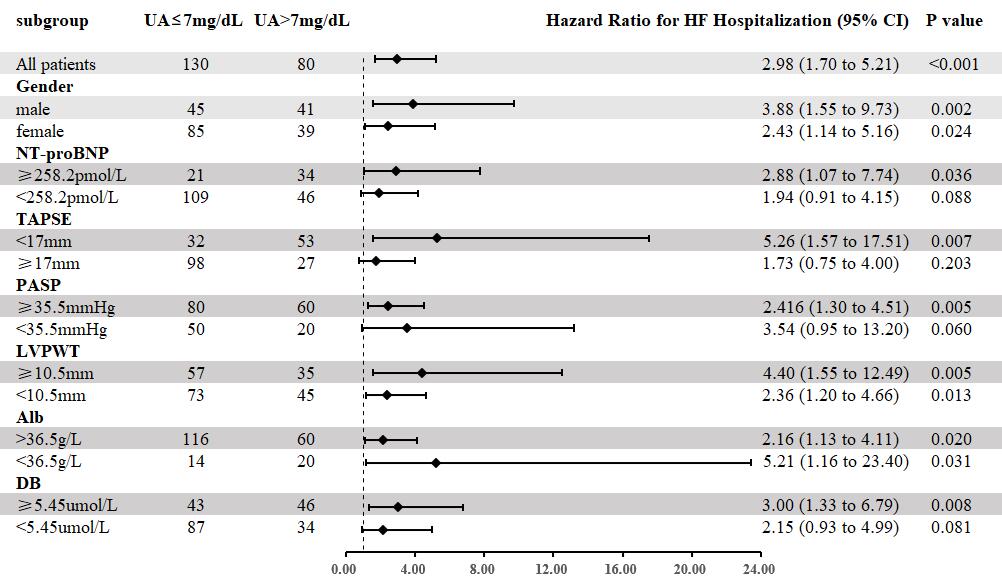


**Figure S3**. Cox regression analysis of serum UA levels on heart failure readmission in different subgroups. Alb = albumin; DB = direct bilirubin; LVPW = left ventricular posterior wall; PASP = pulmonary artery systolic blood pressure; TAPSE = tricuspid annular plane systolic excursion; UA =uric acid.

**Table** S**1.** Multivariate binary logistic regression analysis of the effect of UA on the absolute value of right ventricular dysfunction.

|  |  | OR | 95%CI | P-value |
| --- | --- | --- | --- | --- |
| **Model 1** |  |  |  |  |
|  | UA (mg/dL) | 2.161 | 1.693- 2.758 | <0.001 |
|  | IVST (mm) | 1.132 | 0.805- 1.591 | 0.476 |
|  | LVPW (mm) | 0.583 | 0.390- 0.872 | 0.009 |
|  | Diuretics (n) | 1.894 | 0.845- 4.244 | 0.121 |
| **Model 2** |  |  |  |  |
|  | UA (mg/dL) | 1.889 | 1.490- 2.395 | <0.001 |
|  | Atrial Fibrillation | 2.085 | 0.981- 4.433 | 0.056 |
|  | Heart rate (beats/min) | 1.005 | 0.988- 1.022 | 0.599 |
|  | NT-proBNP (pmol/L) | 1.004 | 1.002- 1.006 | 0.001 |
| **Model 3** |  |  |  |  |
|  | UA (mg/dL) | 1.854 | 1.475- 2.331 | <0.001 |
|  | LDL-C (mmol/L) | 0.624 | 0.387- 1.007 | 0.053 |
|  | DB (uml/L) | 1.069 | 0.977- 1.171 | 0.147 |
|  | Alb (umol/L) | 0.922 | 0.833- 1.020 | 0.116 |
| **Model 4** |  |  |  |  |
|  | UA (mg/dL) | 2.028 | 1.564- 2.630 | <0.001 |
|  | LVPW (mm) | 0.698 | 0.524- 0.930 | 0.014 |
|  | NT-proBNP (pmol/L) | 1.004 | 1.001- 1.006 | 0.001 |
|  | Atrial Fibrillation | 1.799 | 0.826- 3.918 | 0.139 |
|  | LDL-C (mmol/L) | 0.632 | 0.383- 1.042 | 0.072 |

Model 1 was adjusted for UA, IVST, LVPW, and Diuretics.

Model 2 was adjusted for UA, Atrial Fibrillation, Heart rate, and NT-proBNP.

Mode l3 was adjusted for UA, LDL-C, DB, and Alb.

Model 4 was adjusted for UA, LVPW, NT-proBNP, Atrial Fibrillation, and LDL-C.

**Table S2.** Univariate and multivariate analyses of heart failure readmission associated with selected baseline variables.

| Parameters | Univariate analysis | | Multivariate analysis | |
| --- | --- | --- | --- | --- |
|  | HR (95%CI) | p-value | HR (95%CI) | p-value |
| High UA | 2.980 (1.704- 5.211) | <0.001 | 3.027 (1.519- 6.031) | 0.002 |
| Age | 0.993 (0.972- 1.015) | 0.536 | 0.989 (0.966- 1.012) | 0.333 |
| Male gender | 1.281 (0.743- 2.208) | 0.374 | 1.140 (0.647- 2.008) | 0.651 |
| NYHA III IV | 1.512 (0.872- 2.622) | 0.141 | 0.925 (0.587- 4.451) | 0.828 |
| CAD | 1.291 (0.749- 2.226) | 0.358 | 0.973 (0.531- 1.785) | 0.930 |
| AF | 1.217 (0.702- 2.112) | 0.484 | 0.992 (0.531- 1.858) | 0.981 |
| RVD | 1.781 (1.030- 3.081) | 0.039 | 0.741 (0.352- 1.561) | 0.431 |
| NT-proBNP | 1.002 (1.001- 1.003) | 0.001 | 1.002 (1.000- 1.004) | 0.010 |
| Cr | 1.006 (0.999- 1.013) | 0.078 | 0.998 (0.989- 1.008) | 0.706 |

AF = atrial fibrillation; CAD = coronary artery disease; Cr = creatinine; DB = direct bilirubin; NT-proBNP = N-terminal B-type natriuretic peptide; PASP = pulmonary artery systolic blood pressure; RVD = right ventricular dysfunction;TAPSE = tricuspid annular plane systolic excursion; TB = total bilirubin; UA = uric acid.
